# Body Mass Index and Mortality in the General Population and in Subjects with Chronic Disease in Korea: A Nationwide Cohort Study (2002-2010)

**DOI:** 10.1371/journal.pone.0139924

**Published:** 2015-10-13

**Authors:** Nam Hoon Kim, Juneyoung Lee, Tae Joon Kim, Nan Hee Kim, Kyung Mook Choi, Sei Hyun Baik, Dong Seop Choi, Rodica Pop-Busui, Yousung Park, Sin Gon Kim

**Affiliations:** 1 Division of Endocrinology and Metabolism, Department of Internal Medicine, Korea University College of Medicine, Seoul, Korea; 2 Department of Biostatistics, Korea University College of Medicine, Seoul, Korea; 3 Department of Statistics, Korea University, Seoul, Korea; 4 Division of Metabolism, Endocrinology, and Diabetes, Department of Internal Medicine, University of Michigan, Ann Arbor, MI, United States of America; Shanghai Institute of Hypertension, CHINA

## Abstract

**Background:**

The association between body mass index (BMI) and mortality is not conclusive, especially in East Asian populations. Furthermore, the association has been neither supported by recent data, nor assessed after controlling for weight changes.

**Methods:**

We evaluated the relationship between BMI and all-cause or cause-specific mortality, using prospective cohort data by the National Health Insurance Service in Korea, which consisted of more than one million subjects. A total of 153,484 Korean adults over 30 years of age without pre-existing cardiovascular disease or cancer at baseline were followed-up until 2010 (mean follow-up period = 7.91 ± 0.59 years). Study subjects repeatedly measured body weight 3.99 times, on average.

**Results:**

During follow-up, 3,937 total deaths occurred; 557 deaths from cardiovascular disease, and 1,224 from cancer. In multiple-adjusted analyses, U-shaped associations were found between BMI and mortality from any cause, cardiovascular disease, and cancer after adjustment for age, sex, smoking status, alcohol consumption, physical activity, socioeconomic status, and weight change. Subjects with a BMI < 23 kg/m^2^ and ≥ 30 kg/m^2^ had higher risks of all-cause and cause-specific mortality compared with the reference group (BMI 23–24.9 kg/m^2^). The lowest risk of all-cause mortality was observed in subjects with a BMI of 25–26.4 kg/m^2^ (adjusted hazard ratio [HR] 0.86; 95% CI 0.77 to 0.97). In subgroup analyses, including the elderly and those with chronic diseases (diabetes mellitus, hypertension, and chronic kidney disease), subjects with a BMI of 25–29.9 kg/m^2^ (moderate obesity) had a lower risk of mortality compared with the reference. However, this association has been attenuated in younger individuals, in those with higher socioeconomic status, and those without chronic diseases.

**Conclusion:**

Moderate obesity was associated more strongly with a lower risk of mortality than with normal, underweight, and overweight groups in the general population of South Korea. This obesity paradox was prominent in not only the elderly but also individuals with chronic disease.

## Introduction

Obesity, generally defined by high body mass index (BMI), is closely associated with incident chronic disease including hypertension, type 2 diabetes and atherosclerotic cardiovascular diseases (CVDs) [1,2]. Previous studies have also shown that high BMI is related to an increased incidence of various types of cancers [3]. A logical assumption that overweight or obesity is associated with higher mortality than that of normal weight, however, is not conclusive. This ‘obesity paradox’ has been widely observed in different race or ethnic groups, and in patients with pre-existing chronic diseases such as hypertension, diabetes mellitus, heart failure, and chronic kidney disease [4–7].

Possible explanations for this paradox, mainly focusing on a study bias and confounding factors, include selection or survivorship bias, unintentional weight loss following chronic disease, treatment bias, and effects of major confounders including smoking status and age [8]. However, even after adjusting these major confounders, the obesity paradox still remains [9].

It has also been observed that the association between BMI and mortality is largely dependent on race. Asians generally have a lower BMI value, but the risk of obesity-related disorders and CVD is quite comparable to that of Europeans, even for those belonging to lower BMI categories [10]. Therefore, The World Health Organization (WHO) suggested lower optimal BMI (18.5–22.9), overweight (23–24.9), moderate obesity (25–29.9), and severe obesity (≥30) categories for Asians than for Europeans [11]. Large epidemiological studies in East Asian populations showed generally U- or J-shaped associations between BMI and mortality, with varying BMI values suggested for the lowest risk of mortality. Jee et al. showed that the lowest mortality occurred in Korean populations with BMI values of 23–24.9 [12]. However, in other studies including East Asians, the BMI values of the lowest risk of death were widely distributed over 18.5–27.5 [13–16]. These studies had more than enough statistical power owing to the large-scale data; however, they also had limitations: inaccurate weight and disease status information obtained by self-reported questionnaires; having large proportions of data missing; lacking an important variable such as socioeconomic status; and most importantly, disregarding body weight changes. Repeated body weight measurement information is particularly critical because of its contribution to study results as a major confounder.

We also note that most studies of East Asians were based on baseline BMI measures from the early 1990s. Since obesity prevalence, disease status, and cause-specific mortality rates are rapidly changing, especially in East Asia [17,18], an association between BMI and risk of mortality is needed to be re-evaluated with recent data, taking into account body weight changes and previously-overlooked variables. In this regard, we analyzed a recently developed Korean National Health Insurance sample cohort database, which includes more than one million subjects, from 2002 to 2010, to evaluate the association between BMI and mortality. We also assessed whether the association is consistent regardless of the subject’s chronic disease status.

## Methods

### Study population

A national health insurance system in Korea has been initiated since 1963 according to the National Health Insurance Act, and it became compulsory for all citizens in South Korea to participate. Currently, the National Health Insurance Service (NHIS) maintains and manages all databases of Korea’s health service utilization. The NHIS is also in charge of the national health examination programs, which include a general health examination for all insured employees or self-employed persons over the age of 40, as well as their dependents; it is recommended that this examination be undertaken at least biennially. Moreover, a life-transition examination (for those reaching milestones of 40 and 66 years of age), a cancer examination, and a pediatric examination (for those between 4 and 71 months post-birth) are also managed by the NHIS.

The NHIS released a National Sample Cohort (2002–2010) database (The NHIS-NSC [2002–2010]) in July 2014, consisting of 1,025,340 Koreans as an initial 2002 cohort, and followed the subjects for 9 years up to 2010. The cohort data represents about 2.2% of the source population in 2002 (46,605,433), sampled systematically to represent an individual’s total annual medical expenses within each of 1,476 strata defined by age, sex, eligibility status (employed or self-employed), and income level (20 quantiles for each eligibility status plus medical aid beneficiary) combinations. This is a semi-dynamically constructed cohort database; namely, the cohort has been followed up to either the time of the participant’s disqualification of health services due to death or emigration, or the end of the study period, while samples of newborn infants are included annually. The database contains eligibility and demographic information regarding health insurance and medical aid beneficiaries, medical bill details, medical treatment and disease histories, as well as prescriptions; such data are constructed after converting insurance claim information to the first day of medical treatment.

In the cohort, the subject’s mortality information along with cause of death was included; the latter was classified according to the 10th revision of the International Classification of Diseases (ICD-10) codes, obtained from the Korean National Statistical Office. Furthermore, the laboratory and survey questionnaire data of general and life-transition health examinations for all cohort members was merged.

From this cohort, we selected subjects who had received health examinations at least once between 2003 and 2004, noting that citizens are recommended to take the examination at least once biennially. We then selected men and women over 30 years of age who measured their own body weight. We did not use the 2002 cohort data in order to clearly identify the subject’s past medical history as well as to avoid possible medical history inaccuracies in the first year. To minimize reverse causality and any effects of pre-existing disease on mortality, we also excluded subjects who had reported CVD or cancer from 2002 to 2004, and further excluded deaths within less than 2 years after the baseline measurement of body weight. As a result, a total of 153,484 subjects were included in our follow-up study.

This study were based on data from NHIS, informed consent was not specifically obtained individually. Data were fully anonymized and de-identified for the analysis. This study was approved by the Institutional Review Board of Korea University Hospital. (IRB number: ED14188)

### Determinants of disease and demographic factors

BMI was calculated as the subject’s weight (kg) divided by height (m^2^), as measured at regular medical check-up programs. Most participants measured their body weight and height at 2–5 year intervals, and the mean (SD) number of measurements taken per subject was 3.99 (2.01).

At the time of weight measurement, systolic and diastolic pressure was also measured; serum samples for fasting glucose, hemoglobin, and total cholesterol levels were also obtained after an overnight fast at each examination site. Detailed histories of smoking status, alcohol consumption, and physical activity (including amount and frequency) were obtained via questionnaire. We conducted statistical analyses using the simplified status classification of smoking (current, former, or never), alcohol (drinker or non-drinker), and physical activity (no activity, ≤2 times/week, or ≥3 times/week). The subjects’ socioeconomic status (SES) was categorized into 3 groups (lowest 30%, middle 40% and highest 30%) based on 20 strata of income levels provided in the NHIS-NSC (2002–2010) database.

The participants’ medical history was identified by a combination of the following: clinic and pharmacy codes of ICD-10, lists of prescribed medicine, and previous medical histories. To identify diabetes mellitus (DM) and hypertension (HTN) status, laboratory data were also used in addition to medical records and health examination survey results based on the criteria of those with fasting serum glucose ≥126 mg/dL for DM, and those with systolic blood pressure ≥140 mmHg or diastolic blood pressure ≥90 mmHg for HTN.

### Statistical analysis

An association between BMI level and mortality risk was analyzed with Cox’s proportional hazard regression model. The BMI level at baseline was categorized into 8 groups from lowest (<18.5 kg/m^2^) to highest (≥32.5 kg/m^2^). The remaining groups were 18.5–19.9, 20–21.4, 21.5–22.9, 23–24.9, 25–26.4, 26.5–27.9, 28–29.9 and 30–32.4 kg/m^2^. These groupings were based on the WHO obesity classification for Asian populations and the previous Korean study (KCPS) [11, 12]. The reference group (23–24.9) was set according to the KCPS, and the population mean of BMI in this NHIS cohort, which was 23.8 kg/m^2^.

The hazard ratio (HR) and 95% confidence interval (CI) for each groups, relative to the reference (BMI 23–24.9 kg/m^2^) were estimated, after adjusting for the subjects’ age, sex, and BMI change relative to his/her initial baseline value. Adjusting for changes in BMI would eliminate the effect on mortality of intentional weight control in healthy subjects or unintentional weight change in those with disease, providing us with a pure BMI effect on mortality. The same analysis was conducted by additionally adjusting for the subjects’ smoking status (current, former or never), alcohol drinking habits (non-drinker, ≤2 times/week or ≥3 times/week), physical activities (no activity, ≤2 times/week or ≥3 times/week), and 20 SES groups. The HRs of BMI on all-cause, cardiovascular, and cancer mortalities were also estimated. Furthermore, in order to evaluate the robustness of the association between BMI and mortality, stratified analyses according to the subjects’ sex, smoking status (lifetime non-smoker, past or current smoker), age group (30–49, 50–69, ≥70 years) and three SES group (lowest 30%, middle 40%, or highest 30%) were performed.

We also examined the association according to the subjects’ status of DM (yes or no), CVD (yes or no), and cancer (yes or no). The interactions between BMI and different stratifications were tested in order to examine differences in HRs across strata. Tukey’s method was used for multiple mean comparisons of different groups or strata. All statistical analyses were performed using SAS version 9.4 (SAS Institute Inc., Cary, NC, USA). All p-values were two-tailed, and values less than 0.05 were considered statistically significant.

## Results

### Study subjects

A total of 153,484 participants at baseline from 2003 to 2004 were followed up to 2010 for a mean duration of 7.91 ± 0.59 years. In that time, a total of 3,937 deaths occurred, with 557 (14.1%) resulting from CVD and 1,224 (31.1%) from cancer. The mean age of all participants at baseline was 47.1 ± 11.8 years, 61.1% of whom were male. Baseline demographic characteristics stratified by BMI categories are presented in Table 1. Trends of mean age across BMI categories differed by sex (p < 0.001). Mean age gradually decreased as BMI level increased in men, whereas it increased with BMI level in women, implying that younger men tend to be obese; in contrast, older women tend towards obesity. The proportion of subjects categorized as current smokers was shown to peak in the lowest BMI group for both sexes. Differential patterns of SES by sex were also observed. The smallest proportion of men belonging to the upper 30% of the SES scale was observed in the lowest BMI group (<18.5 kg/m^2^), while the proportion of the upper 30% was relatively high between 23 and 30 kg/m^2^ BMI categories. However, among women, the smallest proportion of the upper 30% was in the highest BMI group, and the proportion decreased as the BMI group increased from 21.5–22.9 kg/m^2^ to the highest BMI group (≥32.5 kg/m^2^). Meanwhile, systolic blood pressure, fasting serum glucose, and total serum cholesterol increased as the BMI increased. By extension, the risk of incident DM, HTN, and CVD was directly proportional to increasing BMI, as expected (S1 Table).

**Table 1:** Baseline characteristics according to body mass index category.

	BMI (kg/m^2^)										
	<18.5	18.5–19.9	20–21.4	21.5–22.9	23–24.9	25–26.4	26.5–27.9	28–29.9	30–32.4	≥ 32.5	
**Men**											p-value
Number of participants	2,098	4,887	10,812	15,696	26,121	17,005	9,186	5,576	1,846	506	
Age (y), mean (SD)	50.0 (15.4)	46.6 (13.6)	45.8 (12.4)	45.4 (11.7)	45.4 (11.1)	45.2 (10.6)	44.4 (10.4)	43.8 (10.4)	42.3 (10.0)	40.6 (10.6)	<0.001
Weight (kg), mean (SD)	50.0 (4.7)	55.1 (4.4)	59.3 (4.7)	63.6 (4.8)	68.5 (5.2)	73.5 (5.4)	78.1 (5.9)	82.8 (6.3)	89.5 (6.7)	103.4 (20.9)	<0.001
Smoking status (%)											<0.001
Current	57.8	57.7	54.4	50.6	47.8	46.8	47.0	48.6	51.0	57.4	
Former	6.9	6.6	8.2	9.2	10.5	11.0	10.9	10.2	10.2	6.8	
Non	35.3	35.8	37.4	40.2	41.8	42.2	42.1	41.2	38.8	35.7	
Alcohol consumption (%)											<0.001
Non	40.6	36.2	33.2	31.6	29.8	29.6	29.5	29.5	29.7	32.3	
< twice a week	42.6	46.7	49.1	51.4	54.6	53.8	53.9	53.2	53.3	51.6	
≥ three times a week	16.8	17.1	17.8	17.0	15.7	16.6	16.6	17.3	17.1	16.1	
Physical activity (%)											<0.001
Non	65.9	60.7	53.2	49.9	46.0	44.6	44.1	46.0	43.8	46.7	
< twice a week	23.0	26.4	30.5	31.6	33.6	34.4	34.4	33.5	36.1	35.8	
≥ three times a week	11.1	12.9	16.3	18.5	20.4	21.0	21.5	20.5	20.1	17.5	
Socioeconomic status (%)											<0.001
Upper 30%	35.3	37.9	41.9	46.3	49.6	50.5	49.4	47.5	46.7	37.2	
Mid 40%	41.6	42.9	41.2	39.0	36.3	36.4	36.9	38.3	38.2	46.9	
Lower 30%	23.0	19.2	16.9	14.7	14.1	13.2	13.8	14.2	15.1	15.9	
Systolic blood pressure (mmHg), mean (SD)	120.6 (17.3)	121.7 (16.0)	122.8 (16.0)	124.5 (15.7)	126.5 (15.8)	128.4 (15.8)	129.9 (16.0)	132.0 (16.3)	134.3 (16.5)	137.3 (17.7)	<0.001
Glucose (mg/dL), mean (SD)	95.3 (37.8)	94.2 (34.4)	94.6 (31.1)	95.6 (30.8)	96.5 (30.5)	98.1 (31.9)	98.7 (28.7)	100.2 (32.3)	103.1 (32.6)	102.1 (31.5)	<0.001
Total cholesterol (mg/dL), mean (SD)	177.3 (32.8)	180.0 (35.5)	185.9 (34.8)	191.1 (35.0)	197.6 (36.0)	202.6 (37.2)	205.0 (37.5)	208.3 (40.5)	211.2 (38.8)	210.9 (38.4)	<0.001
**Women**											p-value
Number of participants	2,010	4,734	9,679	11,258	14,414	8,116	4,618	3,096	1,402	424	
Age (y), mean (SD)	46.8 (15.8)	45.3 (13.0)	46.7 (12.0)	48.9 (11.4)	51.3 (11.1)	52.7 (10.8)	53.7 (10.7)	54.3 (10.7)	53.6 (10.7)	53.0 (10.7)	<0.001
Weight (kg), mean (SD)	43.0 (4.4)	47.3 (3.9)	50.6 (4.0)	53.8 (4.2)	57.3 (4.4)	61.1 (4.7)	64.5 (4.9)	67.9 (5.2)	73.2 (5.7)	81.0 (9.2)	<0.001
Smoking status (%)											<0.001
Current	5.3	3.2	3.2	2.7	2.3	2.2	2.7	2.2	3.5	4.5	
Former	0.4	0.5	0.4	0.4	0.4	0.4	0.4	0.4	0.4	0.5	
Non	94.4	96.3	96.4	97.0	97.4	97.4	96.9	97.4	96.1	95.0	
Alcohol consumption (%)											<0.001
Non	80.3	76.8	76.9	78.2	79.4	79.4	81.7	81.6	79.2	80.3	
< twice a week	18.4	21.7	21.2	20.1	18.7	18.7	16.8	16.2	17.9	16.4	
≥ three times a week	1.3	1.5	1.8	1.7	2.0	2.0	1.6	2.2	2.9	3.3	
Physical activity (%)											<0.001
Non	78.57	71.5	66.9	64.55	64	64.35	65.52	68.4	68.55	70.14	
< twice a week	12.67	16.61	18.34	18.73	18.03	17.85	16.76	16.32	15.97	14.69	
≥ three times a week	8.76	11.89	14.76	16.72	17.97	17.79	17.72	15.28	15.47	15.17	
Socioeconomic status (%)											<0.001
Upper 30%	35.9	37.9	38.6	39.4	38.7	37.2	36.0	34.0	34.0	28.03	
Mid 40%	37.7	34.0	32.3	30.9	32.3	32.9	34.5	34.9	33.5	38.24	
Lower 30%	26.4	28.2	29.1	29.6	29.0	30.0	29.4	31.1	32.6	33.73	
Systolic blood pressure (mmHg), mean (SD)	114.8 (17.2)	115.5 (16.1)	117.6 (16.2)	120.3 (17.4)	123.6 (18.0)	126.7 (18.4)	129.5 (18.7)	131.5 (18.8)	133.6 (18.2)	137.6 (19.5)	<0.001
Glucose (mg/dL), mean (SD)	89.1 (20.8)	89.8 (25.6)	91.0 (26.1)	92.4 (26.1)	94.3 (29.7)	96.3 (30.9)	98.8 (34.4)	99.2 (31.5)	101.2 (31.1)	103.4 (32.5)	<0.001
Total cholesterol (mg/dL), mean (SD)	182.7 (33.6)	185.3 (35.0)	189.2 (35.5)	195.0 (37.4)	200.3 (38.0)	205.9 (39.9)	207.5 (38.8)	209.5 (40.0)	210.8 (38.4)	212.3 (40.0)	<0.001

Data are presented by mean ± standard deviation (SD) or %.

### Association between body mass index and mortality in the total population

Associations between BMI and all-cause mortality for all participants, stratified by their demographic factors, are presented in Table 2. In age-, sex- and BMI change-adjusted analyses, there was a U-shaped relationship between BMI and all-cause mortality, with the lowest mortality in the BMI group of 25–27.4 kg/m^2^ (HR 0.88; 95% CI 0.80–0.97). The highest risk of mortality was observed in the lowest BMI group (<18.5 kg/m^2^). This association was maintained even after additionally adjusting for subjects’ smoking, alcohol intake, physical activity status, and SES. Further analyses stratified by smoking status and sex also did not alter the associations. There was a trend of increasing risk of mortality in subjects with BMI lower than the reference group. Subjects with a BMI equal to or more than 30 kg/m^2^ (severe obesity group) showed high mortality risk; however, those with a BMI of 25–29.9 kg/m^2^ (moderate obesity group) had lower HRs than normal, underweight, overweight, and severe obesity groups.

**Table 2:** Association between body mass index category and all-cause mortality.

	BMI (kg/m^2^)									
	<18.5	18.5–19.9	20–21.4	21.5–22.9	23–24.9	25–26.4	26.5–27.9	28–29.9	30–32.4	≥ 32.5
**All subjects**										
Number of deaths	329	407	586	716	895	474	255	177	72	26
Age, and sex adjusted HR	2.65	1.91	1.36	1.26	1	0.86	0.87	0.96	1.15	1.26
95% CI	2.33–3.01	1.69–2.14	1.22–1.51	1.14–1.39		0.77–0.96	0.76–1.00	0.82–1.13	0.91–1.47	0.83–1.91
Multivariable adjusted HR	2.31	1.73	1.25	1.23	1	0.86	0.88	0.95	1.12	1.25
95% CI	2.02–2.63	1.53–1.95	1.13–1.40	1.12–1.36		0.77–0.97	0.76–1.02	0.81–1.13	0.88–1.44	0.82–1.89
**Sex**										
Men										
Number of deaths	236	309	409	498	625	329	159	100	37	15
Multivariable adjusted HR	2.37	1.86	1.29	1.24	1	0.86	0.82	0.90	1.09	1.28
95% CI	1.78–3.17	1.41–2.44	1.02–1.62	1.00–1.54		0.67–1.10	0.61–1.11	0.64–1.26	0.66–1.80	0.57–2.91
Women										
Number of deaths	93	98	177	218	270	145	96	77	35	11
Multivariable adjusted HR	2.17	1.42	1.19	1.22	1	0.87	0.98	1.03	1.14	1.19
95% CI	1.70–2.77	1.13–1.80	0.98–1.44	1.02–1.46		0.71–1.07	0.77–1.24	0.79–1.34	0.80–1.64	0.65–2.18
p-interaction (men vs. women)	0.55	0.06	0.50	0.86		0.88	0.26	0.43	0.86	0.86
**Smoking status**										
Non-smoker										
Number of deaths	151	178	304	372	457	262	149	110	49	18
Multivariable adjusted HR	2.49	1.66	1.32	1.28	1	0.93	0.94	1.02	1.21	1.58
95% CI	2.07–3.01	1.39–1.98	1.14–1.53	1.12–1.47		0.80–1.08	0.78–1.13	0.83–1.26	0.90–1.63	0.98–2.54
Current, or former smoker										
Number of deaths	178	229	282	344	438	212	106	67	23	8
Multivariable adjusted HR	2.45	1.86	1.25	1.19	1	0.79	0.80	0.82	0.98	0.75
95% CI	1.90–3.17	1.46–2.35	1.01–1.55	0.98–1.45		0.63–0.99	0.60–1.07	0.59–1.15	0.58–1.65	0.31–1.82
p-interaction (non-smoker vs. current, or former smoker)	0.90	0.36	0.65	0.46		0.17	0.27	0.21	0.43	0.10
**Age**										
30–49 years										
Number of deaths	21	47	113	127	160	103	55	40	18	5
Multivariable adjusted HR	1.38	1.24	1.42	1.22	1	1.04	0.94	1.09	1.39	0.90
95% CI	0.82–2.33	0.85–1.81	1.06–1.91	0.92–1.61		0.77–1.41	0.65–1.38	0.71–1.67	0.77–2.51	0.32–2.56
50–69 years										
Number of deaths	114	170	233	332	451	239	120	95	38	14
Multivariable adjusted HR	2.90	2.01	1.19	1.25	1	0.81	0.78	0.97	1.20	1.22
95% CI	2.35–3.59	1.68–2.41	1.01–1.40	1.08–1.44		0.69–0.95	0.64–0.96	0.77–1.22	0.86–1.68	0.70–2.13
≥70 years										
Number of deaths	194	190	240	257	284	132	80	42	16	7
Multivariable adjusted HR	2.07	1.56	1.22	1.21	1	0.90	1.06	0.84	0.81	1.58
95% CI	1.56–2.75	1.20–2.02	0.96–1.55	0.97–1.52		0.69–1.18	0.76–1.47	0.56–1.27	0.42–1.56	0.63–3.99
p-interaction (30–49 years vs. 50–69 years)	<0.01	0.01	0.24	0.87		0.09	0.34	0.59	0.63	0.57
p-interaction (50–69 years vs. ≥70 years)	0.02	0.06	0.83	0.80		0.41	0.07	0.50	0.24	0.59
**Socioeconomic status**										
Upper 30%										
Number of deaths	123	126	194	242	304	173	96	64	24	7
Multivariable adjusted HR	2.94	2.00	1.44	1.31	1	0.94	0.92	1.03	1.17	1.02
95% CI	2.15–4.02	1.48–2.69	1.12–1.86	1.03–1.66		0.72–1.24	0.66–1.29	0.69–1.52	0.64–2.14	0.39–2.64
Middle 40%										
Number of deaths	106	129	234	252	330	153	92	60	22	12
Multivariable adjusted HR	2.33	1.45	1.30	1.13	1	0.74	0.84	0.90	1.01	1.35
95% CI	1.86–2.93	1.17–1.78	1.10–1.55	0.96–1.34		0.60–0.90	0.66–1.06	0.68–1.19	0.66–1.56	0.74–2.44
Lower 30%										
Number of deaths	100	150	155	220	258	144	66	51	25	7
Multivariable adjusted HR	1.78	1.79	1.01	1.27	1	0.93	0.89	0.93	1.20	1.33
95% CI	1.28–2.47	1.34–2.40	0.77–1.31	0.99–1.63		0.70–1.24	0.62–1.28	0.61–1.41	0.65–2.21	0.51–3.46
p-interaction (upper 30% vs. middle 40%)	0.15	0.03	0.43	0.25		0.08	0.56	0.51	0.64	0.56
p-interaction (middle 40% vs. lower 30%)	0.11	0.15	0.06	0.38		0.11	0.75	0.88	0.58	0.98

In the multivariable adjusted model, data was adjusted for age, sex, smoking status, alcohol intake, physical activity, socioeconomic status, and body weight change. In the analyses of stratified subgroups, the variable used in stratification was excluded.

We note that a stratification of study population by age and SES showed different associations between BMI and all-cause mortality across subgroups (Table 2). In subjects younger than 50 years old, the relationship between BMI and the mortality was attenuated in the lowest BMI group, compared to subjects aged more than 50 years, while there was no significant association in the groups with the other BMI groups. There were similar U-shaped associations for both middle and lower SES, with the lowest all-cause mortality HRs in the moderate obesity group. However, no significant association between high BMI and all-cause mortality was found, especially in subjects with higher SES (upper 30% of the scale).

**Fig 1:**
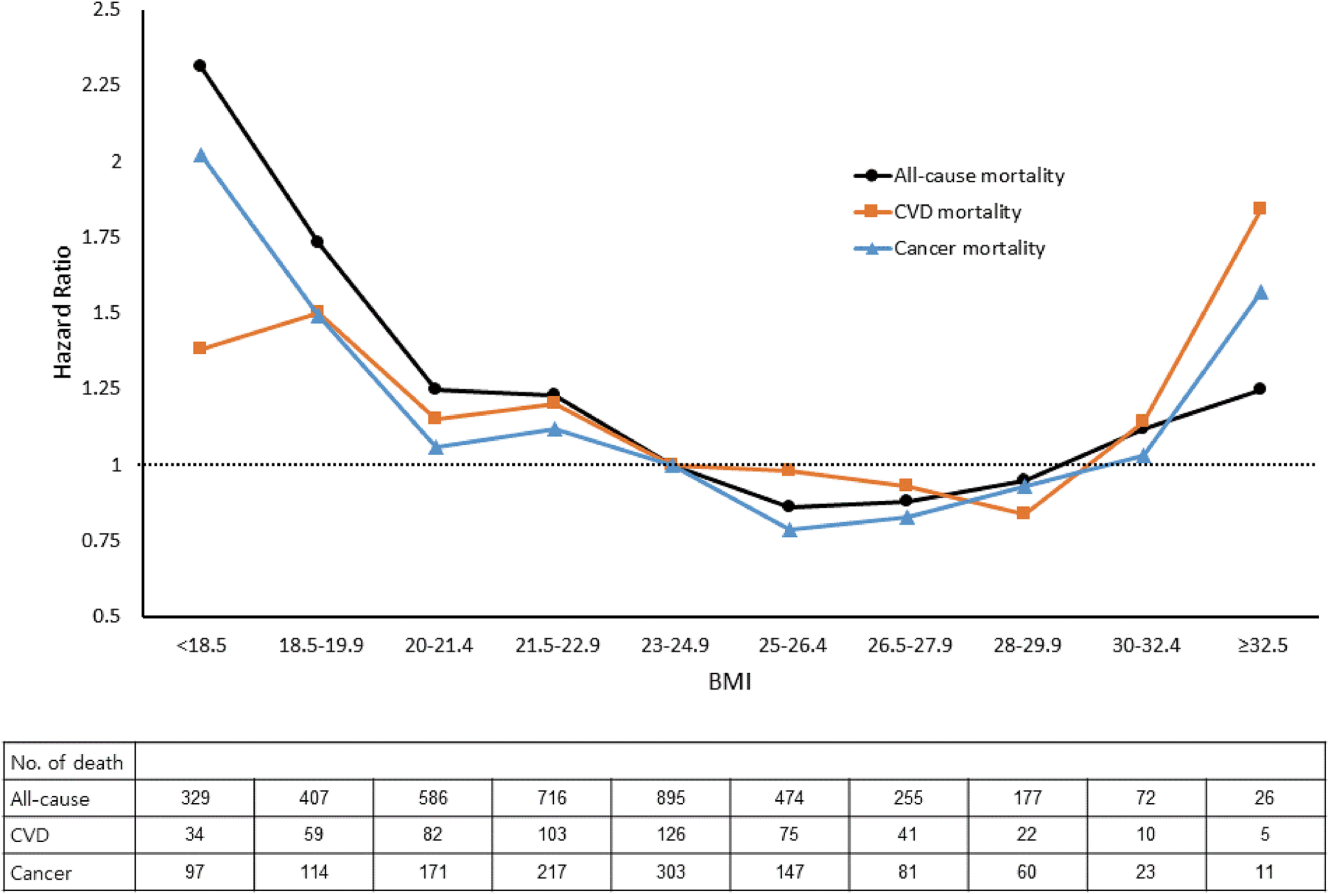
Association between body mass index and all-cause and cause-specific mortality in Korean adults. The analyses were adjusted for age, sex, and weight change.

This U-shaped association between BMI and CVD and cancer mortalities has also been observed (Fig 1; S2 and S3 Tables). Although statistical significance was weakened because of the relatively small sample size, the lowest risk of mortality for CVD or cancer was observed in subjects with BMI values of 25–29.9 kg/m^2^. An elevated risk of mortality from CVD or cancer was also consistent in subjects with lower BMI groups than the reference group as for that of all-cause mortality. A positive association between severe obesity and mortality risk was prominent for cases of CVD mortality.

### Body mass index and mortality in subjects with chronic disease

**Fig 2:**
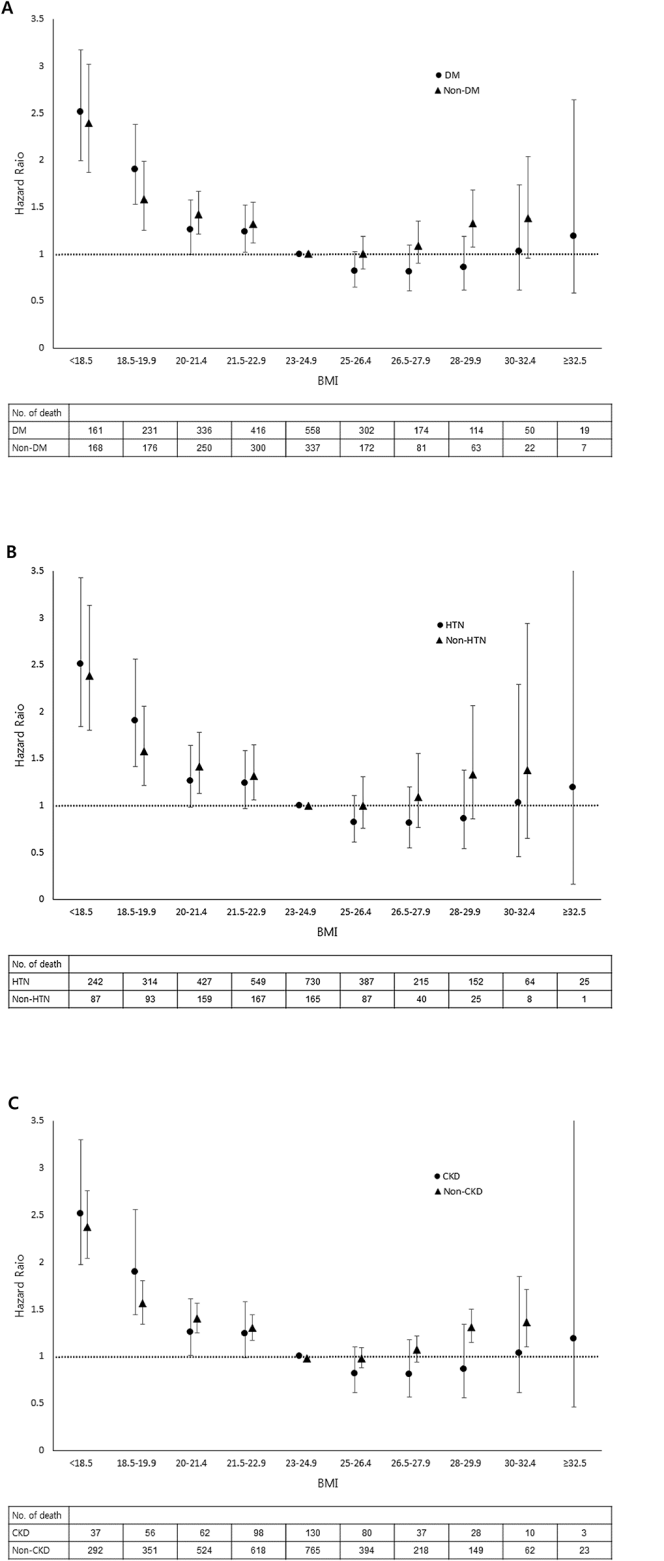
Association between body mass index and all-cause mortality according to disease status. The association between body mass index (BMI) and mortality was presented separately by those presenting with and without prevalent diabetes mellitus (DM) (A), hypertension (HTN) (B), and chronic kidney disease (CKD) (C). All analyses were adjusted for age, sex, smoking status, alcohol intake, physical activity, socioeconomic status, and body weight change.

We assessed an association between BMI and mortality risk within subgroups stratified by chronic medical disease status, which included DM, HTN, and chronic kidney disease (CKD) (Fig 2; S4 Table). There was also a U-shaped association between BMI and all-cause mortality both with and without DM presentation. The highest mortality risk was addressed in subjects with the lowest BMI (<18.5) in both strata. The adjusted HR was lowest in three moderate obesity groups (BMI 25–26.4, 26.5–27.9, and 28–29.9) in DM patients, of which magnitude were smaller than those in subjects without DM. HTN and CKD also showed a similar association. For CVD- and cancer-associated mortalities, nearly identical findings were observed, but with slightly reduced statistical significance because of a small frequency of deaths within the subgroups, compared to the total participants (S5 and S6 Tables).

## Discussion

In this recent population-based, prospective cohort study, we performed comprehensive analyses of the associations between BMI and all-cause mortality as well as cause-specific mortalities. In general, there were U-shaped associations between BMI and all-cause, CVD-, and cancer-associated mortalities in the total subjects. An increased risk of mortality in subjects with BMI of less than 23 and more than 30, compared with the reference range (BMI, 23–24.9) was also found, with the lowest mortality in the moderate obesity group (BMI, 25–29.9), more specifically in the BMI range of 25–26.4. Further subgroup analyses stratified by sex and smoking status did not change the associations, while they were varied across age and SES subgroups.

Previous large epidemiological studies including East Asians showed similar U-shaped associations between BMI and all-cause or cause-specific mortality, but the BMI group with the lowest mortality risk has widely ranged from 18.5 to 27.5 according to study populations and cause of death [12–16]. Among them, the large-scale Korean study (KCPS), which enrolled more than 1 million people from 1992 to 1995, reported that the risk of all-cause mortality was lowest in subjects with a BMI of 23.0–24.9 (overweight group) [12]. Our data, compiled about 10 years after the aforementioned study and comprising members of the general population of South Korea, revealed a more intensified obesity paradox by shifting the lowest mortality risk into BMI of 25–26.4, which demonstrated the decreased mortality risk in obese subjects who had increased risk of many chronic diseases including CVD [19]. Considering the optimal BMI of 18.5–23 as an Asian standard suggested by the WHO, this 10-year change in obesity paradox patterns is an interesting observation that remains to be fully elucidated.

A previous study in the United States encompassing the first to third rounds of the National Health and Nutrition Examination Survey (NHANES) provided an important clue about this phenomenon [20]. They reported that relative risks of mortality associated with obesity continuously decreased from NHANES 1 (1971–1975) to NHANES 3 (1988–1994). Over the past few decades in Korea, life expectancy dramatically increased from 65.7 years in 1980 to 76.0 years in 2000 [21]. During the same period, there were serial changes in general population mortality causes; deaths from CVD decreased from 28.3% to 21.2%, while those from cancer increased from 17.2% to 29.1% [22]. We also identified that cardiovascular mortality dropped by about 4%, but cancer mortality did not change from 2002 to 2010 in our dataset (data not shown). Meanwhile, we observed an increased prevalence of obesity compared with that of KCPS; the proportion of subjects having a BMI of ≥25 was 36.4% in men, and 29.5% in women, which is much higher than that of the KCPS (23.7% in men, 26.8% in women). Prevalence of severe obesity (BMI ≥ 30) has also increased from 0.8% and 2.4% to 2.5% and 3.1% in men and women, respectively. However, the prevalence of underweight (BMI < 18) was comparable between both studies (2.2% and 4.7% in our study versus 2.4% and 3.4% in the KCPS, for men and women, respectively). This implies that the prevalence of obesity and obesity-related disorders increased during the past decade in South Korea, but mortality risk from CVD has decreased and life expectancy has increased, possibly by means of improved medical care, which synthetically can lead to a decreased mortality in obese individuals.

It was also of interest to note that associations between BMI, and CVD and cancer mortalities were, to a certain degree, different each other; the HR of cancer mortality more rapidly decreased in the moderate obesity group than that of CVD mortality. This indicated that high BMI may represent an optimal physical and nutritional state for protecting against catabolic diseases such as cancer. Growing evidence suggests that low muscle mass is a poor prognostic indicator of mortality, and BMI is positively correlated with muscle mass or lean body mass, especially in the elderly [23,24]. This suggests that the association between BMI and mortality differs between age groups, as shown in our study that lower mortality risk associated with obesity was more pronounced in older (≥50 years old) than in younger (30–49 years old) age groups. Many previous, large epidemiological studies, such as NHANES and KCPS, have already proven similar findings; excess mortality risk associated with obesity was weakened in elderly subjects [12,25]. However, this also indicates a limitation of utilizing BMI as an indicator of obesity because BMI cannot accurately discriminate fat from muscle mass. The European Prospective Investigation into Cancer and Nutrition (EPIC) study showed this limitation of BMI by revealing a strong association of waist circumference with mortality risk after adjusting BMI [26]. Similar arguments for health-related quality of life within the general Korean population can also be found [27]. Since we did not have information related to body composition for our participants, this remains a study limitation.

Another important finding in our study was that SES was revealed as a major determinant of the association between BMI and mortality. First of all, we would like to mention that the identification of study subjects’ SES was almost complete because the Korean national health care system covers all citizens, and an individual`s medical insurance premium is directly proportional to their income. We found that the effect of SES on BMI differed by sex. Although there were inverse U-shaped associations between BMI and SES in both sexes, the main difference among them was that men in the lowest social class were prone to being underweight, while women in a low social class were likely to have a high BMI. It is well known that SES influences not only accessibility to a medical care system but also life expectancy; in a study of mortality, it needs to be taken into account as a major confounder, as our study reflects. Alter et al. showed that those in higher income groups were associated with an increased rate of access to coronary angiography and revascularization for acute myocardial infarction, as well as decreased mortality, compared with those in lower income groups [28]. Another study also argued that SES was associated with excess mortalities for ischemic heart disease and diabetes in black women [29]. As shown in Table 2, however, our data demonstrate that no significant relationship existed between moderate to severe obesity and all-cause mortality in subjects with high income (upper 30% of SES). We therefore suspect that, even though one is severely obese, his/her mortality risk could be comparable with those belonging to the reference BMI group if he/she is able to easily access and be insured by a medical care system.

We also found that the association between BMI and mortality varied according to the state of chronic diseases, including DM, HTN, and CKD; the obesity paradox was more profound in patients with chronic diseases than those without. Previous large amounts of data have demonstrated the paradox in patients with chronic diseases. As stated above, some authors have explained the obesity paradox by focusing on a study bias, or confounding factors. For example, most epidemiologic studies that investigated the effect of BMI on mortality excluded subjects with pre-existing CVD, and thus obese CVD patients who were at high risk of death were not included in the analysis, obscuring the actual relationship between obesity and mortality [8]. Another interpretation focused on treatment bias; Schenkeveld et al. showed that obesity paradox was attenuated after adjusting for optimal medical treatment, which explained the improved outcome in the obese group [30]. An age effect has also been frequently discussed; Lainscak found that many large-scale studies of the obesity paradox included elderly subjects, suggesting that the beneficial effect of a high BMI was limited to specific age groups [31].

Alternately, several efforts also have been made to overcome a hidden bias, which resulted in the paradox. For example, in the case of diabetes, since no clear cut exists to identify whether weight loss was unintentional due to development of diabetes or intentional after diagnosis, we suggested the weight change as a major confounder in this analysis. Recently, there were two large-scale, yet conflicting studies published, both of which included patients with incident diabetes based on initial body weight at the time of diabetic diagnosis [5,32]. Therefore, the associations found between BMI and mortality in our study cannot be directly compared with those of previous studies, as our study included patients with prevalent diabetes (not limited to those with incident diabetes). However, we took steps to ensure meaningful data: we carefully defined diabetes using multiple sources of information including laboratory data; tried to minimize effects of reverse causality by excluding patients with established CVDs or cancers at baseline, as well as those who died within less than 2 years after baseline measurements; and adjusted the subjects’ weight change in the analysis. Similar results were found in other studies conducted mainly with European patients [33,34], but our study extended to patients with HTN and CKD, who had little change in body weight at the time of disease diagnosis. Additionally, when the analysis was extended to patients with DM, CVD, or cancer, even the severe obesity group (BMI ≥ 32.5 kg/m^2^) had a lower HR of mortality than the reference group, suggesting a protective role for obesity in patients with various morbidities (S1 Fig).

We found that weight change was a major confounder in associations between BMI and all-cause, CVD, and cancer-related mortality. Mortality risks for groups with a BMI less than the reference value in the model without change of BMI as a control variable were lower than when BMI change was included, and this difference in risk increased as BMI decreased. Conversely, for groups with a BMI higher than the reference, the mortality risks without change of BMI were greater than when BMI change was included, and the difference in risk between the two models increased with increasing BMI. Thus, excluding BMI change in the association between BMI and mortality made it harder to determine the true extent of the obesity paradox (S7 Table).

There are several strengths in our study. The NHIS-NSC (2002–2010) cohort consists of representative samples of about one million Koreans who were prospectively followed during the study period. We were able to obtain accurate information with regard to critical variables to measure an effect of BMI on mortality risk at baseline as well as during follow-up; BMI was calculated by directly measured body weight and height, and the subjects’ disease status was identified by using health examination survey questionnaires, disease codes, and laboratory or anthropometric data. We were also able to collect detailed histories of smoking, alcohol consumption, physical activity, and SES. Most of all, by considering a major criticism of previous studies regarding the obesity paradox, we incorporated BMI changes with repeated measured body weight in the analyses in order to overcome a reversal causality between unintentional or intentional weight loss and mortality.

A limitation of this study includes a potential selection bias. The NHIS recommends, but does not obligate all health insurance subscribers to undergo at least once biennial health examination. Therefore, this cohort probably includes healthier individuals, or those who are more concerned about their health. Another limitation is the lack of confounders such as marital and educational status, and body composition, as discussed previously.

In this study, we identified several novel findings regarding the association between BMI and mortality. The BMI level with the lowest risk of mortality was changed; that is, a shift to the right compared with the Korean data from 10 years ago (from 23–24.9 to 25.0–27.5 kg/m^2^). This suggests that the change of age, combined with social structure, could alter the associations between obesity and health status. Interestingly, the association observed in this study resembles that of developed countries [20]. The WHO classification of obesity for Asians published in the year 2000 suggested that the optimal BMI was 18.5–23 kg/m^2^. This was largely based on the relationship between BMI and the incidence of obesity-related disorders. However, the “optimal” BMI should take into account mortality as well as morbidity. Furthermore, changes in the epidemiologic characteristics of Asian populations over the last decade, including an increasing prevalence of obesity, should also be considered. The findings of our study likewise suggest a higher optimal BMI for Asians. Moreover, our data suggests that the obesity paradox needs to be interpreted separately, according to individuals’ demographic and health status; this was apparent for older persons and patients with chronic diseases. Health professionals and providers may need to take a different approach to patients to maintain optimal BMI, by understanding and taking into consideration a variety of health status factors.

## Supporting Information

S1 Fig Association between body mass index and all-cause mortality according to DM, CVD, and cancer status. (DOCX)

S1 STROBE Checklist STROBE Checklist. (DOCX)

S1 Table Association between body mass index category and incidence of chronic diseases. (DOCX)

S2 Table Association between body mass index category and cardiovascular disease mortality. (DOCX)

S3 Table Association between body mass index category and cancer mortality. (DOCX)

S4 Table Association between body mass index category and all-cause mortality according to disease status. (DOCX)

S5 Table Association between body mass index category and cardiovascular disease mortality according to disease status. (DOCX)

S6 Table Association between body mass index category and cancer mortality according to disease status. (DOCX)

S7 Table Association between body mass index category and all-cause mortality, NOT adjusting for weight changes. (DOCX)

## Abbreviations

BMI: body mass index
CVD: cardiovascular disease
WHO: World Health Organization
ICD: International Classification of Diseases
DM: diabetes mellitus
HTN: hypertension
CKD: chronic kidney disease
SES: socioeconomic status
