# Supplementary material for: Body Mass Index and Mortality in the General Population and in Subjects with Chronic Disease in Korea: A Nationwide Cohort Study (2002-2010)
Source: PLoS One. 2015 Oct 13;10(10):e0139924. doi: 10.1371/journal.pone.0139924 (PMC4604086; doi:10.1371/journal.pone.0139924)
Supplement: S1 Fig — (DOCX) [file pone.0139924.s001.docx]

**S1 Fig. Association between body mass index and all-cause mortality according to DM, CVD, and cancer status.**


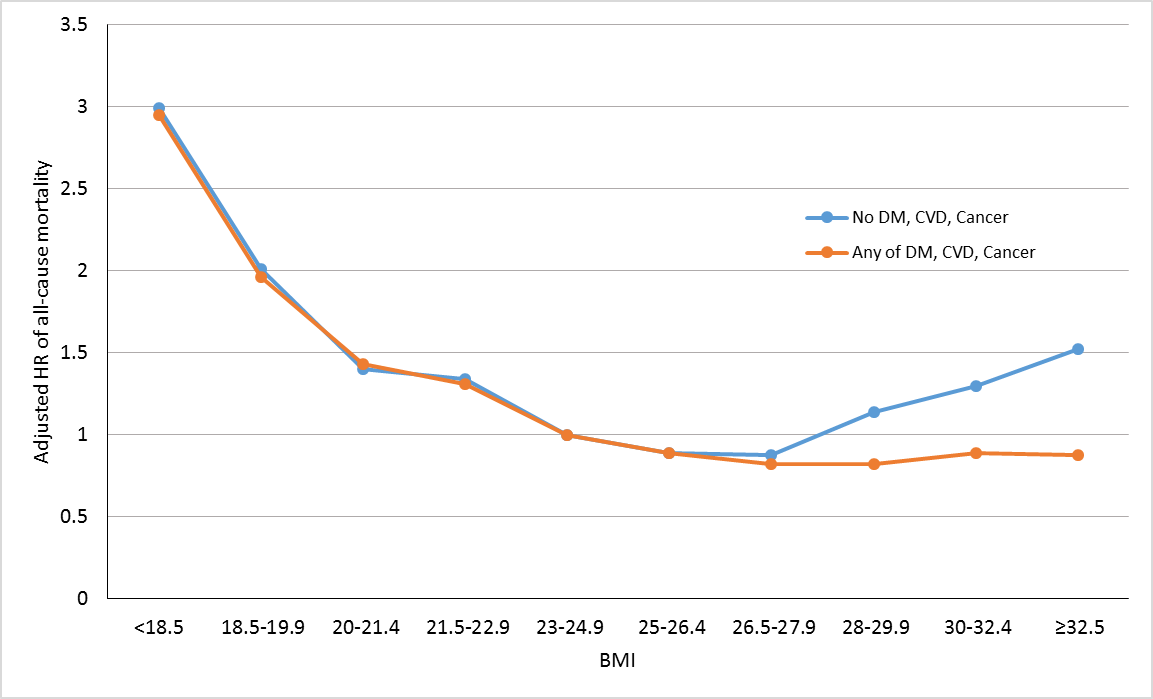


Analyses were adjusted for age, sex, smoking status, alcohol intake, physical activity, socioeconomic status, and body weight change.
