# Supplementary material for: Body Mass Index and Mortality in the General Population and in Subjects with Chronic Disease in Korea: A Nationwide Cohort Study (2002-2010)
Source: PLoS One. 2015 Oct 13;10(10):e0139924. doi: 10.1371/journal.pone.0139924 (PMC4604086; doi:10.1371/journal.pone.0139924)
Supplement: S1 Table — (DOCX) [file pone.0139924.s003.docx]

**S1 Table. Association between body mass index category and incidence of chronic diseases**

|  | BMI (kg/m^2^) | | | | | | | |
| --- | --- | --- | --- | --- | --- | --- | --- | --- |
|  | <18.5 | 18.5–20.4 | 20.5–22.9 | 23–24.9 | 25–27.4 | 27.5–29.9 | 30–32.4 | ≥32.5 |
| **Incident DM** |  |  |  |  |  |  |  |  |
| Adjusted HR | 0.68 | 0.73 | 0.86 | 1 | 1.22 | 1.47 | 1.77 | 2.09 |
| 95% CI | 0.64–0.72 | 0.70–0.76 | 0.83–0.88 |  | 1.19–1.25 | 1.42–1.52 | 1.68–1.87 | 1.91–2.28 |
| **Incident HTN** |  |  |  |  |  |  |  |  |
| Adjusted HR | 0.53 | 0.63 | 0.78 | 1 | 1.25 | 1.60 | 1.93 | 2.34 |
| 95% CI | 0.50–0.56 | 0.61–0.64 | 0.77–0.80 |  | 1.22–1.27 | 1.56–1.63 | 1.85–2.01 | 2.18–2.52 |
| **Incident CVD** |  |  |  |  |  |  |  |  |
| Adjusted HR | 0.66 | 0.77 | 0.83 | 1 | 1.13 | 1.30 | 1.40 | 1.73 |
| 95% CI | 0.61–0.72 | 0.73–0.80 | 0.81–0.86 |  | 1.09–1.16 | 1.24–1.35 | 1.31–1.50 | 1.54–1.95 |
| **Incident cancer** |  |  |  |  |  |  |  |  |
| Adjusted HR | 0.95 | 0.98 | 0.99 | 1 | 0.98 | 0.95 | 0.89 | 0.85 |
| 95% CI | 0.89–1.00 | 0.94–1.01 | 0.97–1.02 |  | 0.96–1.01 | 0.92–0.99 | 0.3–0.95 | 0.74–0.96 |

In the adjusted model, data was adjusted for age, sex, and body weight change.

BMI, body mass index; DM, diabetes mellitus; HR, hazard ratio; CI, confidence interval; HTN, hypertension; CVD, cardiovascular disease.
