# Supplementary material for: Body Mass Index and Mortality in the General Population and in Subjects with Chronic Disease in Korea: A Nationwide Cohort Study (2002-2010)
Source: PLoS One. 2015 Oct 13;10(10):e0139924. doi: 10.1371/journal.pone.0139924 (PMC4604086; doi:10.1371/journal.pone.0139924)
Supplement: S2 Table — (DOCX) [file pone.0139924.s004.docx]

**S2 Table. Association between body mass index category and cardiovascular disease mortality**

|  | BMI (kg/m^2^) | | | | | | | | | |
| --- | --- | --- | --- | --- | --- | --- | --- | --- | --- | --- |
|  | <18.5 | 18.5 – 19.9 | 20 – 21.4 | 21.5 – 22.9 | 23 – 24.9 | 25 – 26.4 | 26.5 – 27.9 | 28 – 29.9 | 30 – 32.4 | ≥ 32.5 |
| **All subjects** |  |  |  |  |  |  |  |  |  |  |
| Number of deaths | 34 | 59 | 82 | 103 | 126 | 75 | 41 | 22 | 10 | 5 |
| Adjusted HR | 1.38 | 1.50 | 1.15 | 1.20 | 1 | 0.98 | 0.93 | 0.84 | 1.14 | 1.84 |
| 95% CI | 0.92-2.08 | 1.08-2.08 | 0.86-1.53 | 0.92-1.57 |  | 0.73-1.31 | 0.64-1.36 | 0.53-1.34 | 0.60-2.17 | 0.73-4.63 |
| **Sex** |  |  |  |  |  |  |  |  |  |  |
| Men |  |  |  |  |  |  |  |  |  |  |
| Number of deaths | 17 | 44 | 51 | 71 | 85 | 46 | 23 | 12 | 5 | 3 |
| Adjusted HR | 0.94 | 1.50 | 1.05 | 1.20 | 1 | 0.88 | 0.83 | 0.77 | 1.19 | 2.33 |
| 95% CI | 0.41-2.11 | 0.73-3.05 | 0.58-1.92 | 0.67-2.12 |  | 0.48-1.61 | 0.39-1.78 | 0.30-1.97 | 0.33-4.36 | 0.37-14.9 |
| Women |  |  |  |  |  |  |  |  |  |  |
| Number of deaths | 17 | 15 | 31 | 32 | 41 | 29 | 18 | 10 | 5 | 2 |
| Adjusted HR | 2.36 | 1.49 | 1.36 | 1.20 | 1 | 1.20 | 1.13 | 0.97 | 1.16 | 1.52 |
| 95% CI | 1.31-4.26 | 0.82-2.71 | 0.84-2.21 | 0.75-1.94 |  | 0.74-1.95 | 0.63-2.03 | 0.48-1.95 | 0.46-2.96 | 0.37-6.31 |
| p-interaction (men vs. women) | 0.03 | 0.99 | 0.40 | 0.98 |  | 0.32 | 0.43 | 0.63 | 0.97 | 0.65 |
| **Smoking status** |  |  |  |  |  |  |  |  |  |  |
| Non-smoker |  |  |  |  |  |  |  |  |  |  |
| Number of deaths | 18 | 29 | 42 | 48 | 59 | 45 | 24 | 12 | 8 | 4 |
| Adjusted HR | 1.98 | 2.03 | 1.33 | 1.28 | 1 | 1.26 | 1.19 | 0.87 | 1.52 | 2.65 |
| 95% CI | 1.15-3.41 | 1.30-3.18 | 0.89-1.98 | 0.87-1.87 |  | 0.85-1.86 | 0.74-1.91 | 0.47-1.63 | 0.72-3.18 | 0.96-7.35 |
| Current, or former smoker |  |  |  |  |  |  |  |  |  |  |
| Number of deaths | 16 | 30 | 40 | 55 | 67 | 30 | 17 | 10 | 2 | 1 |
| Adjusted HR | 1.21 | 1.35 | 1.12 | 1.16 | 1 | 0.73 | 0.78 | 0.87 | 0.60 | 0.83 |
| 95% CI | 0.55-2.64 | 0.72-2.54 | 0.63-1.96 | 0.68-1.96 |  | 0.41-1.32 | 0.38-1.64 | 0.35-2.17 | 0.12-2.92 | 0.09-8.06 |
| p-interaction (non-smoker vs. current , or former smoker) | 0.22 | 0.21 | 0.55 | 0.71 |  | 0.07 | 0.27 | 0.99 | 0.25 | 0.32 |
| **Age** |  |  |  |  |  |  |  |  |  |  |
| 30–49 years |  |  |  |  |  |  |  |  |  |  |
| Number of deaths | 1 | 4 | 12 | 15 | 20 | 12 | 8 | 3 | 1 | 2 |
| Adjusted HR | 0.56 | 0.86 | 1.11 | 1.17 | 1 | 0.92 | 1.04 | 0.71 | 0.62 | - |
| 95% CI | 0.07-4.73 | 0.26-2.82 | 0.47-2.64 | 0.53-2.57 |  | 0.39-2.18 | 0.38-2.89 | 0.18-2.83 | 0.07-5.47 | - |
| 50–69 years |  |  |  |  |  |  |  |  |  |  |
| Number of deaths | 9 | 24 | 32 | 41 | 56 | 34 | 18 | 12 | 6 | 0 |
| Adjusted HR | 1.82 | 1.99 | 1.30 | 1.19 | 1 | 0.92 | 0.96 | 0.91 | 1.48 | - |
| 95% CI | 0.90-3.70 | 1.20-3.29 | 0.84-2.02 | 0.79-1.80 |  | 0.60-1.42 | 0.56-1.63 | 0.48-1.74 | 0.64-3.44 | - |
| ≥70 years |  |  |  |  |  |  |  |  |  |  |
| Number of deaths | 24 | 31 | 38 | 47 | 50 | 29 | 15 | 7 | 3 | 3 |
| Adjusted HR | 1.27 | 1.27 | 1.01 | 1.20 | 1 | 1.15 | 0.87 | 0.84 | 0.98 | - |
| 95% CI | 0.53-3.06 | 0.64-2.53 | 0.54-1.88 | 0.67-2.14 |  | 0.61-2.18 | 0.37-2.02 | 0.30-2.33 | 0.23-4.14 | - |
| p-interaction (30-49 years vs. 50-69 years) | 0.28 | 0.17 | 0.72 | 0.95 |  | 0.99 | 0.87 | 0.73 | 0.43 | - |
| p-interaction (50-69 years vs. ≥70 years) | 0.42 | 0.20 | 0.42 | 0.99 |  | 0.49 | 0.82 | 0.87 | 0.58 | - |

In the adjusted model, data was adjusted for age, sex, and body weight change.

In the analyses stratified subgroups, the variable used in stratification was excluded.

BMI, body mass index; HR, hazard ratio; CI, confidence interval.
