# Supplementary material for: Body Mass Index and Mortality in the General Population and in Subjects with Chronic Disease in Korea: A Nationwide Cohort Study (2002-2010)
Source: PLoS One. 2015 Oct 13;10(10):e0139924. doi: 10.1371/journal.pone.0139924 (PMC4604086; doi:10.1371/journal.pone.0139924)
Supplement: S3 Table — (DOCX) [file pone.0139924.s005.docx]

**S3 Table. Association between body mass index category and cancer mortality**

|  | BMI (kg/m^2^) | | | | | | | | | |
| --- | --- | --- | --- | --- | --- | --- | --- | --- | --- | --- |
|  | <18.5 | 18.5 – 19.9 | 20 – 21.4 | 21.5 – 22.9 | 23 – 24.9 | 25 – 26.4 | 26.5 – 27.9 | 28 – 29.9 | 30 – 32.4 | ≥ 32.5 |
| **All subjects** |  |  |  |  |  |  |  |  |  |  |
| Number of deaths | 97 | 114 | 171 | 217 | 303 | 147 | 81 | 60 | 23 | 11 |
| Adjusted HR | 2.02 | 1.49 | 1.06 | 1.12 | 1 | 0.79 | 0.83 | 0.93 | 1.03 | 1.57 |
| 95% CI | 1.59-2.58 | 1.20-1.86 | 0.87-1.29 | 0.94-1.34 |  | 0.64-0.96 | 0.65-1.07 | 0.70-1.24 | 0.66-1.61 | 0.82-3.02 |
| **Sex** |  |  |  |  |  |  |  |  |  |  |
| Men |  |  |  |  |  |  |  |  |  |  |
| Number of deaths | 80 | 85 | 123 | 146 | 220 | 100 | 54 | 29 | 13 | 6 |
| Adjusted HR | 2.34 | 1.55 | 1.10 | 1.06 | 1 | 0.74 | 0.80 | 0.70 | 1.01 | 1.42 |
| 95% CI | 1.28-4.25 | 0.94-2.55 | 0.71-1.70 | 0.72-1.55 |  | 0.48-1.14 | 0.47-1.37 | 0.39-1.26 | 0.41-2.47 | 0.40-5.06 |
| Women |  |  |  |  |  |  |  |  |  |  |
| Number of deaths | 17 | 29 | 48 | 71 | 83 | 47 | 27 | 31 | 10 | 5 |
| Adjusted HR | 1.28 | 1.37 | 0.98 | 1.28 | 1 | 0.91 | 0.90 | 1.34 | 1.08 | 1.77 |
| 95% CI | 0.97-1.68 | 1.10-1.70 | 0.81-1.18 | 1.09-1.51 |  | 0.75-1.09 | 0.72-1.12 | 1.08-1.66 | 0.77-1.51 | 1.11-2.81 |
| p-interaction (men vs. women) | 0.05 | 0.63 | 0.60 | 0.33 |  | 0.36 | 0.67 | 0.03 | 0.89 | 0.73 |
| **Smoking status** |  |  |  |  |  |  |  |  |  |  |
| Non-smoker |  |  |  |  |  |  |  |  |  |  |
| Number of deaths | 31 | 42 | 80 | 112 | 157 | 86 | 45 | 40 | 14 | 7 |
| Adjusted HR | 1.55 | 1.16 | 1.00 | 1.15 | 1 | 0.90 | 0.85 | 1.07 | 1.04 | 1.85 |
| 95% CI | 1.05-2.29 | 0.82-1.64 | 0.76-1.32 | 0.90-1.47 |  | 0.69-1.18 | 0.61-1.18 | 0.75-1.53 | 0.60-1.79 | 0.86-3.96 |
| Current, or former smoker |  |  |  |  |  |  |  |  |  |  |
| Number of deaths | 66 | 72 | 91 | 105 | 146 | 61 | 36 | 20 | 9 | 4 |
| Adjusted HR | 2.81 | 1.80 | 1.21 | 1.11 | 1 | 0.68 | 0.79 | 0.70 | 1.20 | 1.13 |
| 95% CI | 1.73-4.56 | 1.15-2.81 | 0.83-1.78 | 0.78-1.58 |  | 0.46-1.02 | 0.48-1.31 | 0.38-1.28 | 0.50-2.85 | 0.30-4.28 |
| p-interaction (non-smoker vs. current , or former smoker) | 0.02 | 0.05 | 0.33 | 0.86 |  | 0.18 | 0.79 | 0.17 | 0.75 | 0.47 |
| **Age** |  |  |  |  |  |  |  |  |  |  |
| 30–49 years |  |  |  |  |  |  |  |  |  |  |
| Number of deaths | 6 | 6 | 25 | 34 | 41 | 27 | 13 | 14 | 5 | 1 |
| Adjusted HR | 1.46 | 0.66 | 1.19 | 1.32 | 1 | 1.06 | 0.94 | 1.27 | 1.50 | 0.69 |
| 95% CI | 0.54-3.96 | 0.26-1.63 | 0.66-2.12 | 0.78-2.21 |  | 0.60-1.85 | 0.46-1.92 | 0.59-2.71 | 0.50-4.46 | 0.08-5.82 |
| 50–69 years |  |  |  |  |  |  |  |  |  |  |
| Number of deaths | 42 | 61 | 85 | 116 | 180 | 86 | 42 | 37 | 13 | 7 |
| Adjusted HR | 2.64 | 1.85 | 1.06 | 1.10 | 1 | 0.71 | 0.66 | 0.96 | 1.06 | 1.50 |
| 95% CI | 1.86-3.75 | 1.37-2.49 | 0.81-1.38 | 0.87-1.39 |  | 0.55-0.93 | 0.47-0.94 | 0.67-1.37 | 0.60-1.86 | 0.66-3.40 |
| ≥70 years |  |  |  |  |  |  |  |  |  |  |
| Number of deaths | 49 | 47 | 61 | 67 | 82 | 34 | 26 | 9 | 5 | 3 |
| Adjusted HR | 1.87 | 1.43 | 1.08 | 1.12 | 1 | 0.83 | 1.25 | 0.62 | 0.66 | 2.48 |
| 95% CI | 1.13-3.11 | 0.90-2.29 | 0.70-1.67 | 0.74-1.69 |  | 0.51-1.36 | 0.71-2.22 | 0.27-1.40 | 0.18-2.39 | 0.61-10.1 |
| p-interaction (30-49 years vs. 50-69 years) | 0.24 | 0.03 | 0.70 | 0.49 |  | 0.17 | 0.34 | 0.47 | 0.53 | 0.48 |
| p-interaction (50-69 years vs. ≥70 years) | 0.18 | 0.28 | 0.94 | 0.92 |  | 0.53 | 0.03 | 0.29 | 0.48 | 0.48 |

In the adjusted model, data was adjusted for age, sex, and body weight change.

In the analyses stratified subgroups, the variable used in stratification was excluded.

BMI, body mass index; HR, hazard ratio; CI, confidence interval.
