# Supplementary material for: Body Mass Index and Mortality in the General Population and in Subjects with Chronic Disease in Korea: A Nationwide Cohort Study (2002-2010)
Source: PLoS One. 2015 Oct 13;10(10):e0139924. doi: 10.1371/journal.pone.0139924 (PMC4604086; doi:10.1371/journal.pone.0139924)
Supplement: S4 Table — (DOCX) [file pone.0139924.s006.docx]

**S4 Table. Association between body mass index category and all-cause mortality according to disease status**

|  | BMI (kg/m^2^) | | | | | | | | | |
| --- | --- | --- | --- | --- | --- | --- | --- | --- | --- | --- |
|  | <18.5 | 18.5 – 19.9 | 20 – 21.4 | 21.5 – 22.9 | 23 – 24.9 | 25 – 26.4 | 26.5 – 27.9 | 28 – 29.9 | 30 – 32.4 | ≥ 32.5 |
| **DM** |  |  |  |  |  |  |  |  |  |  |
| No. of deaths | 161 | 231 | 336 | 416 | 558 | 302 | 174 | 114 | 50 | 19 |
| Multivariable adjusted HR | 2.22 | 1.73 | 1.30 | 1.21 | 1 | 0.80 | 0.79 | 0.81 | 0.98 | 1.02 |
| 95% CI | 1.70-2.89 | 1.36-2.21 | 1.04-1.62 | 0.99-1.49 |  | 0.63-1.01 | 0.59-1.08 | 0.57-1.14 | 0.57-1.69 | 0.42-2.47 |
| **Non-DM** |  |  |  |  |  |  |  |  |  |  |
| No. of deaths | 168 | 176 | 250 | 300 | 337 | 172 | 81 | 63 | 22 | 7 |
| Multivariable adjusted HR | 2.92 | 1.92 | 1.32 | 1.33 | 1 | 0.91 | 0.89 | 1.08 | 1.12 | 1.32 |
| 95% CI | 2.40-3.55 | 1.59-2.32 | 1.11-1.57 | 1.13-1.56 |  | 0.75-1.10 | 0.70-1.15 | 0.82-1.43 | 0.70-1.78 | 0.61-2.85 |
| p-interaction (DM vs. Non-DM) | 0.04 | 0.41 | 0.89 | 0.38 |  | 0.28 | 0.45 | 0.09 | 0.63 | 0.57 |
| **HTN** |  |  |  |  |  |  |  |  |  |  |
| No. of deaths | 242 | 314 | 427 | 549 | 730 | 387 | 215 | 152 | 64 | 25 |
| Multivariable adjusted HR | 2.51 | 1.90 | 1.26 | 1.24 | 1 | 0.82 | 0.81 | 0.86 | 1.03 | 1.19 |
| 95% CI | 1.84-3.43 | 1.42-2.56 | 0.98-1.64 | 0.97-1.59 |  | 0.61-1.11 | 0.55-1.20 | 0.54-1.38 | 0.46-2.29 | 0.16-8.88 |
| **Non-HTN** |  |  |  |  |  |  |  |  |  |  |
| No. of deaths | 87 | 93 | 159 | 167 | 165 | 87 | 40 | 25 | 8 | 1 |
| Multivariable adjusted HR | 2.39 | 1.58 | 1.42 | 1.32 | 1 | 1.00 | 1.09 | 1.33 | 1.38 | 0.56 |
| 95% CI | 1.81-3.14 | 1.22-2.06 | 1.13-1.78 | 1.06-1.65 |  | 0.76-1.31 | 0.77-1.56 | 0.86-2.07 | 0.65-2.95 | 0.08-4.06 |
| p-interaction (HTN vs. Non-HTN) | 0.75 | 0.22 | 0.38 | 0.61 |  | 0.20 | 0.14 | 0.07 | 0.47 | 0.46 |
| **CKD** |  |  |  |  |  |  |  |  |  |  |
| No. of deaths | 37 | 56 | 62 | 98 | 130 | 80 | 37 | 28 | 10 | 3 |
| Multivariable adjusted HR | 1.63 | 1.59 | 0.90 | 1.01 | 1 | 0.75 | 0.74 | 0.82 | 0.81 | 1.04 |
| 95% CI | 1.09-2.42 | 1.13-2.25 | 0.65-1.25 | 0.76-1.35 |  | 0.55-1.03 | 0.50-1.11 | 0.52-1.30 | 0.40-1.63 | 0.31-3.56 |
| **Non-CKD** |  |  |  |  |  |  |  |  |  |  |
| No. of deaths | 292 | 351 | 524 | 618 | 765 | 394 | 218 | 149 | 62 | 23 |
| Multivariable adjusted HR | 2.50 | 1.78 | 1.33 | 1.26 | 1 | 0.85 | 0.88 | 0.94 | 1.12 | 1.27 |
| 95% CI | 2.17-2.89 | 1.56-2.02 | 1.18-1.49 | 1.13-1.40 |  | 0.75-0.96 | 0.75-1.03 | 0.78-1.13 | 0.86-1.47 | 0.81-1.97 |
| p-interaction (CKD vs. Non-CKD) | 0.03 | 0.54 | 0.02 | 0.14 |  | 0.45 | 0.41 | 0.57 | 0.36 | 0.76 |

In the multivariable adjusted model, data was adjusted for age, sex, smoking status, alcohol intake, physical activity, socioeconomic status, and body weight change.

In the analyses stratified subgroups, the variable used in stratification was excluded. BMI, body mass index; HR, hazard ratio; CI, confidence interval.
