# Supplementary material for: Body Mass Index and Mortality in the General Population and in Subjects with Chronic Disease in Korea: A Nationwide Cohort Study (2002-2010)
Source: PLoS One. 2015 Oct 13;10(10):e0139924. doi: 10.1371/journal.pone.0139924 (PMC4604086; doi:10.1371/journal.pone.0139924)
Supplement: S7 Table — (DOCX) [file pone.0139924.s009.docx]

**S7 Table. Association between body mass index category and all-cause mortality, NOT adjusting for weight changes.**

|  | BMI (kg/m^2^) | | | | | | | | | |
| --- | --- | --- | --- | --- | --- | --- | --- | --- | --- | --- |
|  | <18.5 | 18.5 – 19.9 | 20 – 21.4 | 21.5 – 22.9 | 23 – 24.9 | 25 – 26.4 | 26.5 – 27.9 | 28 – 29.9 | 30 – 32.4 | ≥ 32.5 |
| **All-cause mortality** |  |  |  |  |  |  |  |  |  |  |
| Number of deaths | 329 | 407 | 586 | 716 | 895 | 474 | 255 | 177 | 72 | 26 |
| Age, and sex adjusted HR | 2.57 | 1.87 | 1.34 | 1.25 | 1 | 0.87 | 0.89 | 0.99 | 1.19 | 1.65 |
| 95% CI | 2.26-2.92 | 1.66-2.10 | 1.21-1.49 | 1.13-1.38 |  | 0.78-0.97 | 0.77-1.02 | 0.84-1.16 | 0.93-1.51 | 1.11-2.43 |
| Multivatiable adjusted HR | 2.24 | 1.69 | 1.24 | 1.22 | 1 | 0.87 | 0.89 | 0.98 | 1.16 | 1.62 |
| 95% CI | 1.96-2.56 | 1.50-1.91 | 1.11-1.38 | 1.11-1.35 |  | 0.77-0.97 | 0.77-1.03 | 0.83-1.15 | 0.90-1.48 | 1.09-2.39 |
|  |  |  |  |  |  |  |  |  |  |  |
| **CVD mortality** |  |  |  |  |  |  |  |  |  |  |
| Number of deaths | 34 | 59 | 82 | 103 | 126 | 75 | 41 | 22 | 10 | 5 |
| Age, and sex adjusted HR | 1.68 | 1.83 | 1.29 | 1.26 | 1 | 0.99 | 1.02 | 0.87 | 1.16 | 2.20 |
| 95% CI | 1.15-2.47 | 1.34-2.50 | 0.98-1.71 | 0.97-1.64 |  | 0.74-1.32 | 0.72-1.46 | 0.55-1.37 | 0.61-2.20 | 0.90-5.39 |
| Multivatiable adjusted HR | 1.36 | 1.48 | 1.14 | 1.19 | 1 | 0.98 | 0.94 | 0.85 | 1.16 | 2.07 |
| 95% CI | 0.91-2.04 | 1.07-2.06 | 0.86-1.52 | 0.91-1.56 |  | 0.73-1.31 | 0.65-1.37 | 0.54-1.35 | 0.61-2.21 | 0.85-5.08 |
|  |  |  |  |  |  |  |  |  |  |  |
| **Cancer mortality** |  |  |  |  |  |  |  |  |  |  |
| Number of deaths | 97 | 114 | 171 | 217 | 303 | 147 | 81 | 60 | 23 | 11 |
| Age, and sex adjusted HR | 2.27 | 1.55 | 1.16 | 1.12 | 1 | 0.79 | 0.83 | 0.99 | 1.13 | 2.07 |
| 95% CI | 1.80-2.86 | 1.25-1.93 | 0.96-1.40 | 0.94-1.33 |  | 0.65-0.97 | 0.65-1.06 | 0.75-1.30 | 0.74-1.72 | 1.13-3.78 |
| Multivatiable adjusted HR | 1.96 | 1.46 | 1.05 | 1.11 | 1 | 0.79 | 0.85 | 0.95 | 1.07 | 2.09 |
| 95% CI | 1.54-2.50 | 1.17-1.82 | 0.86-1.27 | 0.93-1.33 |  | 0.65-0.97 | 0.66-1.09 | 0.71-1.27 | 0.68-1.66 | 1.14-3.81 |

In the multivariable adjusted model, data was adjusted for age, sex, smoking status, alcohol intake, physical activity, and socioeconomic status.
